# Regulation of PD-L1 Expression by Nuclear Receptors

**DOI:** 10.3390/ijms24129891

**Published:** 2023-06-08

**Authors:** Yoshimitsu Kiriyama, Hiromi Nochi

**Affiliations:** 1 Kagawa School of Pharmaceutical Sciences, Tokushima Bunri University, Tokushima 769-2193, Kagawa, Japan; nochi@kph.bunri-u.ac.jp; 2 Institute of Neuroscience, Tokushima Bunri University, Tokushima 769-2193, Kagawa, Japan

**Keywords:** PD-L1, nuclear receptor, androgen receptor, estrogen receptor, peroxisome-proliferator-activated receptor, retinoic-acid-related orphan receptor

## Abstract

The suppression of excessive immune responses is necessary to prevent injury to the body, but it also allows cancer cells to escape immune responses and proliferate. Programmed cell death 1 (PD-1) is a co-inhibitory molecule that is present on T cells and is the receptor for programmed cell death ligand 1 (PD-L1). The binding of PD-1 to PD-L1 leads to the inhibition of the T cell receptor signaling cascade. PD-L1 has been found to be expressed in many types of cancers, such as lung, ovarian, and breast cancer, as well as glioblastoma. Furthermore, PD-L1 mRNA is widely expressed in normal peripheral tissues including the heart, skeletal muscle, placenta, lungs, thymus, spleen, kidney, and liver. The expression of PD-L1 is upregulated by proinflammatory cytokines and growth factors via a number of transcription factors. In addition, various nuclear receptors, such as androgen receptor, estrogen receptor, peroxisome-proliferator-activated receptor γ, and retinoic-acid-related orphan receptor γ, also regulate the expression of PD-L1. This review will focus on the current knowledge of the regulation of PD-L1 expression by nuclear receptors.

## 1. Introduction

Immunity is a system that recognizes foreign substances, such as pathogenic microorganisms and cancer cells, as “non-self” and eliminates them from the body. For the immune system to function properly, the activation of the immune response is necessary to eliminate “non-self” cells, in addition to the suppression of the immune response to prevent damage to “self” cells caused by an excessive immune response. T cells play an important role in the specific immune response against cancer cells. T cell activation requires antigen presentation by antigen-presenting cells and co-signaling through the interaction of molecules expressed on the antigen-presenting cells and the T cells. Among these molecules expressed on the T cell surface, those that activate the T cells are called co-stimulatory molecules, which transmit activation signals, while those that inhibit the T cells are called co-repressive molecules, which transmit inactivation signals. T cells receive the antigen presentation information from the antigen-presenting cells via major histocompatibility complexes (MHCs), and T cells make contact with the MHCs molecules on the antigen-presenting cells via the T cell receptor (TCR) and the cluster of differentiation 4/8 (CD4/8), which facilitates antigen recognition [1]. When co-stimulatory molecules on T cells bind to ligands on the antigen-presenting cells, the T cells are activated, and when the co-inhibitory molecules on T cells bind to the ligands on the antigen-presenting cells, T cell activity is suppressed. In this immune cell regulatory mechanism, the function of suppressing an excessive immune response is necessary to prevent injury to the body, but it also allows cancer cells to escape from the immune response and proliferate [2]. Programmed cell death 1 (PD-1) is a co-inhibitory molecule on T cells and an immune checkpoint molecule. The receptors for PD-1 are programmed cell death ligand 1 (PD-L1) and PD-L2 [3,4,5]. The binding of PD-1 and PD-L1 leads to the inhibition of the TCR signaling cascade. PD-L1 mRNA is widely expressed in normal peripheral tissues, including the heart, skeletal muscle, placenta, lungs, thymus, spleen, kidney, and liver. However, PD-L1 mRNA is not detectable in the brain, colon, and small intestine by Northern blot hybridization [5,6]. In addition, PD-L1 has also been found to be expressed in many types of cancers, including melanoma, glioblastoma, and renal, lung, ovarian, and breast cancer [7]. Thus, the inhibition of PD-1 and/or PD-L1 by antibodies is an immunotherapy strategy for cancer treatment [8]. The expression of PD-L1 is upregulated by proinflammatory cytokines, such as interferon-γ, tumor necrosis factor-α, and interleukin-6 (IL-6), and growth factors, such as epidermal growth factor and hepatocyte growth factor, as well as gases, such as oxygen (hypoxia) and nitric oxide, via several transcription factors [9,10,11]. However, various nuclear receptors also regulate the expression of PD-L1. This review focuses on the current knowledge of the regulation of PD-L1 expression by nuclear receptors.

## 2. PD-L1 and Its Distribution

**Figure 1:**
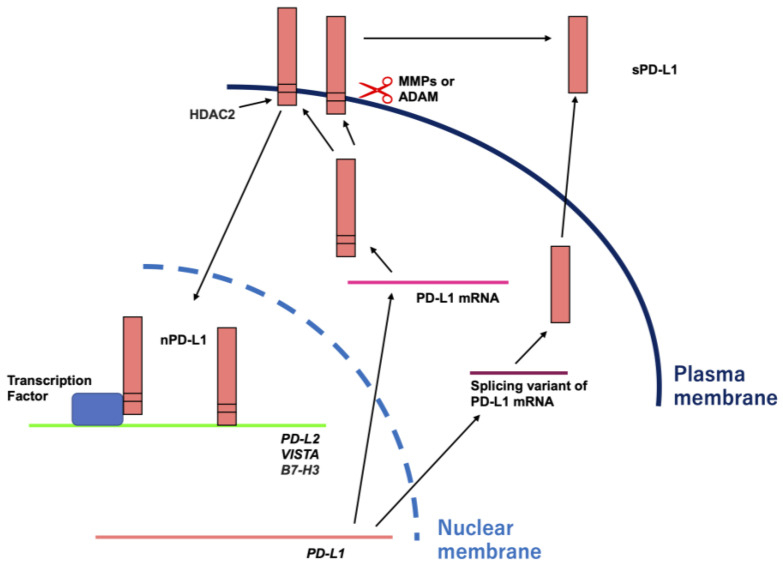
Distribution of PD-L1. PD-L1 in the extracellular spaces is called soluble PD-L1 (sPD-L1). sPD-L1 is produced through the proteolytic cleavage of membrane-bound PD-L1 or alternate splicing of the PD-L1 gene. Proteolytic cleavage of membrane-bound PD-L1 is performed by matrix metalloproteinases (MMPs) or A disintegrin and metalloproteases (ADAMs). Several different alternate splicing variants encode for sPD-L1. PD-L1 in the nucleus is called nuclear PD-L1 (nPD-L1). After membrane-bound PD-L1 is translocated to the nucleolus by histone deacetylase 2 (HDAC2), nPD-L1 binds to DNA not only directly but also via transcription factors, such as NF-κB, IRFs, SP1, and STAT3, leading to the induction of their target genes, such as *PD-L2*, *V-domain Ig suppressor of T cell activation (VISTA)*, and *B7-H3*.

PD-L1, also called B7-H1 or CD274, is a member of the B7 family checkpoint proteins. Members of the B7 family checkpoint proteins consist of B7-1 (also known as CD80), B7-2 (also known as CD86), PD-L1, B7-DC (also known as PD-L2 or CD273), B7-H2 (also known as inducible T cell co-stimulator ligand or CD275), B7-H3 (also known as CD276), B7-H4 (also known as V-set domain containing T cell activation inhibitor 1 (VTCN1), B7x, or B7S1), B7-H5 (also known as V-set immunoregulatory receptor (VISIR), V-domain Ig suppressor T cell activation, (VISTA), GI24, or PD-1H), butyrophilin-like protein 2 (BTNL2), B7-H6 (also known as natural killer cell cytotoxicity receptor 3 ligand 1(NCR3LG1)), and B7-H7 (also known as HERV-H LTR-associating 2 (HHLA2)). The B7 family checkpoint proteins are expressed on antigen-presenting cells and act as ligands for their receptors. Receptors for members of the B7 family checkpoint proteins are expressed on immune cells and have been identified, although some members of the B7 family checkpoint proteins remain to be identified. The B7 family checkpoint proteins and their receptors are expressed on a variety of cells and play a crucial role in the regulation of homeostasis and immune responses, such as inflammation, infection, cancer, and autoimmunity [12]. PD-L1 is a well-known transmembrane protein which is expressed on the membrane surface of many different types of cells, including immune and cancer cells. However, recent studies have demonstrated that PD-L1 functions not only on the membrane surface, but also extracellularly and in the nucleus (Figure 1).

PD-L1 located on the plasma membrane is the well-characterized form of PD-L1. The structure of PD-L1 consists of an extracellular immunoglobulin variable-like domain (Ig-V-domain) and immunoglobulin constant-like domain (Ig-C domain), a transmembrane domain, and an intracellular domain [3,13,14]. PD-L1 expressed on the plasma membrane of antigen-presenting cells interacts with the PD-1 expressed on the T cell plasma membrane. The interaction of PD-L1 with PD-1 activates the PD-1 signaling pathway, inducing the inhibition of the TCR signaling pathway in T cells [15]. When antigen-presenting cells present peptide-loaded major histocompatibility complexes (pMHCs), the T cells recognize these pMHCs through their TCRs. After the recognition of pMHCs by TCRs, the lymphocyte-specific protein tyrosine kinase (Lck), which is a tyrosine kinase belonging to the Src family, interacts with the CD4 or CD8 coreceptors, and the T cell is activated [16]. Lck then phosphorylates the tyrosine residues in the immunoreceptor tyrosine-based activation motif (ITAM) of the intracellular region of CD3, which forms a complex with the TCR [17]. The phosphorylation of the CD3 ITAM leads to the recruitment of the zeta-chain-associated protein kinase 70 kDa (ZAP70), a spleen tyrosine kinase (Syk) family kinase, to the TCR-CD3 complex. This process induces the phosphorylation of ZAP70 [18]. The phosphorylated and activated ZAP70 further phosphorylates the linker for the activation of T cells (LAT) [19]. This LAT phosphorylation recruits downstream signaling molecules, such as phospholipase Cγ1, growth factor receptor-bound protein 2, and lymphocyte cytosolic protein 2, also known as SLP76, and leads to the activation of the TCR signaling cascade and induction of various cytokines [20]. After PD-L1 interacts with PD-1, the PD-1 is phosphorylated by Src kinase in two signaling motifs of the intracellular region of PD-1: the immunoreceptor tyrosine-based inhibitory motif and immunoreceptor tyrosine-based switch motif (ITSM) [21]. The phosphorylated ITSM recruits and activates the Src homology 2 (SH2) domains of SH2-containing phosphatase 2 (SHP2) [22,23]. The activated SHP-2 dephosphorylates signaling molecules, such as ZAP70, which were phosphorylated and activated by the TCR activation [24].

Soluble forms of PD-L1 (sPD-L1) are located in the extracellular space. sPD-L1 is produced through the proteolytic cleavage of the membrane-bound form of PD-L1 or the alternate splicing of the *PD-L1* gene. Proteolytic cleavage of the membrane-bound PD-L1 is performed by matrix metalloproteinases (MMPs) or A disintegrin and metalloproteases (ADAMs) at the stalk domain between the IgC-like domain and transmembrane domain [25]. Among the MMPs, MMP-7, MMP-9, MMP-10, and MMP-13 may cleave the membrane-bound PD-L1 [26,27,28]. Treatment with recombinant MMP-7, MMP-9, or MMP-10 increases the generation of sPD-L1 [28]. In addition, treatment with recombinant MMP-9 or MMP-13 decreased the membrane-bound form of PD-L1 [26,28]. Moreover, treatment with the MMP Inhibitor II, which is an MMP inhibitor, or anti-MMP-10 monoclonal antibodies increased the membrane-bound form of PD-L1 and decreased sPD-L1 [28]. Treatment with CL82198, which is an inhibitor of MMP-13, increased the membrane-bound form of PD-L1 [26,27]. Among the ADAMs, ADAM10 and ADAM17 can cleave membrane-bound PD-L1. TAPI-0, which is an inhibitor of ADAM17 and MMPs, and GI254023X, which is an inhibitor of ADAM10, prevented the generation of sPD-L1. In addition, TAPI-2, which is an inhibitor of ADAMs and MMPs, increased the membrane-bound form of PD-L1. Furthermore, treatment with siRNA against ADAM10 or ADAM17 increased the membrane-bound form of PD-L1. In contrast, treatment with recombinant ADAM10 or ADAM17 reduced the membrane-bound form of PD-L1 [29,30]. The *PD-L1* gene is encoded by seven exons and full-length PD-L1 is synthesized [31]. However, several different alternate splicing variants are generated [32,33] and sPD-L1 is also encoded by alternate splicing [34,35,36,37]. Levels of sPD-L1 are increased in patients with various diseases, such as cancer, sepsis, and viral infections [38,39,40,41,42,43]. Although the physiological and pathophysiological functions of sPD-L1 have not been fully elucidated, sPD-L1 suppresses the activation of T cells by binding to PD-1, in a similar manner to membrane-bound PD-L1 [33,44]. In addition, sPD-L1 generated from a splice variant of PD-L1 mRNA derived from the human placenta possesses a unique domain at the C-terminus, and this domain consists of 18 amino acids including a cysteine residue. sPD-L1 forms a homodimer through a disulfide bond with the cysteine at the C-terminus. Homodimerized sPD-L1 is more effective than the extracellular domain of sPD-L1 in inhibiting T cell proliferation and IFN-γ production [34]. The sPD-L1 levels are also elevated in the blood of pregnant women, and these elevated sPD-L1 levels continue throughout pregnancy [45,46,47]. In addition, these increased levels of sPD-L1 decrease to normal levels postpartum [46,47]. Therefore, it is considered that the increase in sPD-L1 levels in blood during gestation may be associated with the suppression of the maternal immune reaction against the placenta and fetus [46].

Nuclear PD-L1 (nPD-L1) is located in the nucleus and functions to induce various genes [48,49]. PD-L1 has five lysine residues in its cytoplasmic domain, of which lysine 263 is acetylated by histone acetyltransferase p300. In contrast, the acetylated lysine 263 in the PD-L1 cytoplasmic domain is deacetylated by histone deacetylase 2 (HDAC2) [50]. Several molecules, such as huntingtin interacting protein 1-related, adaptin β2, and importin α1, might be involved in nuclear translocation after the deacetylation of PD-L1 by HDAC2 [50]. However, the details of the mechanism of nuclear translocation upon the deacetylation of PD-L1 are unknown. In the nucleus, nPD-L1 binds to genome DNA and regulates various genes, including the genes related to the interferon signaling pathway and immune checkpoint genes, such as *PD-L2*, *VISTA*, and *B7-H3*. In addition, treatment with a combination of anti-PD-1 and HDAC2 inhibitors significantly reduced tumor growth and improved survival compared to anti-PD-1 treatment alone [50]. Furthermore, PD-L1 interacts with several transcription factors. PD-L1 associates with the nuclear factor kappa B (NF-κB) p65 subunit, also known as RelA, and interferon regulatory factors (IRFs), such as IRF3, IRF4, IRF6, and IRF8. These transcription factors are responsible for the regulation of proinflammatory cytokine production [50]. Moreover, PD-L1 interacts with karyopherin subunit beta 1 (KPNB1), which is also known as importin beta 1. KPNB1 plays a crucial role in the nuclear translocation of PD-L1. In addition, PD-L1 interacts with SP1 and elevates the promoter activity and gene expression of *growth arrest-specific 6 (Gas6)* gene. Gas6 activates the c-mer proto-oncogene tyrosine kinase and results in cell proliferation [51]. Hypoxia evokes the phosphorylation of signal transducer and activator of transcription 3 (STAT3) at Y705. Phosphorylated STAT3 interacts with PD-L1, leading to the nuclear translocation of PD-L1 via importins. nPD-L1 with the phosphorylated STAT3 induces the promoter activity and gene expression of *gasdermin C (GSDMC)*. GSDMC plays an important role in the induction of pyroptosis [52].

## 3. Nuclear Receptors

Nuclear receptors play crucial roles in a variety of biological functions, such as differentiation, metabolism, and immune responses, by binding to genomic DNA, leading to the regulation of the expression of their target genes. The nuclear receptor superfamily is composed of 48 members in humans. The activities of most of the nuclear receptor superfamily members are regulated by small lipophilic molecules, such as sex hormones, steroids, and retinoids. However, some members of the nuclear receptor superfamily are orphan nuclear receptors for which ligands have not been found [53,54].

The basic structure of most nuclear receptors consists of an N-terminal domain (NTD), DNA-binding domain (DBD), hinge, and ligand-binding domain (LBD). The NTD has an activator function-1 (AF-1) region (formerly called the A/B domain). The AF-1 region is the region that is highly diverse among the nuclear receptors and interacts with the various co-regulatory proteins. In addition, the AF-1 region is involved in transcription activation in a ligand-independent manner. The DBD is located in the center of the nuclear receptor and binds to the genomic DNA. The DBD has high homology between nuclear receptors and consists of two zinc finger subdomains, which interact with the genomic DNA. In addition, some nuclear receptors, such as liver receptor homolog-1 and germ cell nuclear factor, have a DBD C-terminal extension which allows for further contact with genomic DNA. Short heterodimeric partner (SHP) and dosage-sensitive sex reversal-adrenal hypoplasia congenital critical region on the X chromosome, gene 1 (DAX1), lack the NTD and DBD regions. The hinge region is a linkage region between the DBD and LBD of a nuclear receptor and has the greatest diversity in sequence and size among the nuclear receptors. The LBD is the region that binds to the ligand and interacts with the co-regulatory proteins. The structure of the LBD depends on the type of nuclear receptor. Thus, nuclear receptors regulate the transcriptional activity of genomic DNA by binding to a wide variety of different ligands [54,55].

Nuclear receptors can be classified based on their molecular phylogeny or nuclear receptor characteristics. In the classification based on molecular phylogeny, the nuclear receptors are classified into seven subfamilies (NR1, NR2, NR3, NR4, NR5, NR6, and NR0) [54]. The NR1 family consists of thyroid-hormone-like nuclear receptors. The NR2 family consists of retinoid-X receptor (RXR)-like nuclear receptors. The NR3 family consists of estrogen receptor (ER)-like nuclear receptors. The NR4 family consists of nerve growth factor-induced-B (NGFI-B)-like nuclear receptors. The NR5 family consists of steroidogenic-factor-1-like nuclear receptors. The NR6 family consists of germ cell nuclear factors. The NR0 family consists of atypical nuclear receptors, namely SHP and DAX1. In the classification based on the nuclear receptor characteristics, nuclear receptors are classified into four groups (Class I–VI) [55,56]. Class I consists of steroid receptors, including the androgen receptor (AR), ER, and glucocorticoid receptor (GR). Class II consists of RXR heterodimers, including peroxisome-proliferator-activated receptors (PPARs) and the vitamin D receptor (VDR). Class III consists of homomeric orphan receptors, including RXR and chicken ovalbumin upstream promoter transcription factors (COUP-TFs). Class IV consists of monomeric orphan receptors, including NGFI-B and retinoic-acid-related orphan receptors (RORs).

## 4. Regulation of PD-L1 Expression by Nuclear Receptors

**Figure 2:**
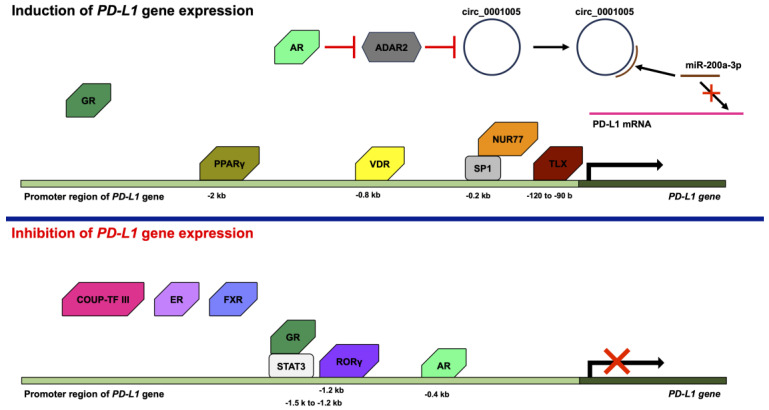
Effects of nuclear receptors on PD-L1 expression. Nuclear receptors positively or negatively regulate the *PD-L1* gene expression. The androgen receptor (AR), glucocorticoid receptor (GR), NUR77, peroxisome-proliferator-activated receptor γ (PPARγ), TLX (homolog of the Drosophila tailless gene), and vitamin D receptor (VDR) induce the *PD-L1* gene expression. AR decreases expression of adenosine deaminases, which act on RNA 2 (ADAR2), leading to the increases in the expression of circ_0001005. Circ_0001005 increases the expression of PD-L1 by competitive binding to microRNA-200a-3p (miR-200a-3p). GR is associated with the induction of *PD-L1* gene expression. NUR77 binds to the −0.2 kb promoter region of the human *PD-L1* gene with SP1. PPARγ binds to the −2 kb promoter region of the human *PD-L1* gene. TLX binds to the −120 to −90 b promoter region of the human *PD-L1* gene. VDR binds to the -0.8 kb promoter region of the human *PD-L1* gene. COUP-TF III, estrogen receptor (ER), farnesoid X receptor (FXR), and retinoic-acid-related orphan receptor γ (RORγ) inhibit the *PD-L1* gene expression. Furthermore, AR and GR also inhibit the *PD-L1* gene expression. AR inhibits the *PD-L1* gene expression by binding to the −0.4 kb promoter region of the human *PD-L1* gene. COUP-TF III, ER, and FXR are associated with the inhibition of *PD-L1* gene expression. GR binds to the −1.5 k to −1.2 kb promoter region of the human *PD-L1* gene with signal transducer and activator of transcription 3 (STAT3). RORγ binds to the −1.2 kb promoter region of the human *PD-L1* gene.

Various transcription factors, such as activator protein 1 (AP-1), NF-κB, STAT1, STAT3, IRF1, IRF3, and hypoxia-inducible factor 1, are activated by a variety of stimuli, such as growth factors, cytokines, and hypoxia, to regulate the gene expression of *PD-L1* [49]. However, *PD-L1* gene expression is not only controlled by transcription factors but also various nuclear receptors (Figure 2).

AR is a member of the steroid hormone nuclear receptor group that consists of ER, GR, the mineralocorticoid receptor, and the progesterone receptor [55,56,57]. Although AR was found to be a ligand-activated nuclear receptor, AR also functions in a ligand-independent manner [58,59,60]. The overexpression of AR results in the decreased expression of PD-L1, and the knockdown of AR by small hairpin RNA (shRNA) leads to the increased expression of PD-L1 in human hepatocellular carcinoma cells. AR binds androgen response element (ARE) in the −0.4 kb promoter region of the human PD-L1 gene. In addition, the anti-PD-L1 antibody treatment of mice implanted with Hep1-6-PCDH (AR-) or Hep1-6-AR (AR+) tumor cells resulted in smaller tumors in mice implanted with Hep1-6-PCDH (AR-) cells than in mice implanted with Hep1-6-AR (AR+) cells [61]. In addition, the treatment of 84E7 cells (AR-expressing thyroid cancer cells) with dihydrotestosterone (DHT), which is an agonist of AR, resulted in the suppression of PD-L1 expression. In contrast, the treatment of 8505C cells (thyroid cancer cells that do not express AR) with DHT did not alter the expression of PD-L1. Treatment of 84E7 cells with Flutamide, a selective AR antagonist, recovered from the DHT-suppressed PD-L1 expression. In addition, AR binds to the PD-L1 gene and downstream of the PD-L1 open reading frame [62]. By contrast, the overexpression of AR resulted in an increase in the expression of PD-L1, and the knockdown of AR by shRNA led to a decrease in the expression of PD-L1 in bladder cancer cells. The increase in the expression of PD-L1 by AR is dependent on adenosine deaminases, which act on RNA 2 (ADAR2), and circ_0001005, which is a circular RNA (circRNA). AR decreases the expression of ADAR2, and the knockdown of ADAR2 by shRNA leads to the increases in the expression of circ_0001005. Circ_0001005 increases the expression of PD-L1 by competitive binding to microRNA-200a-3p (miR-200a-3p) [63]. The circRNA is a single-stranded RNA that forms a covalently linked loop between its 5′ and 3′ ends. circRNA is possibly generated by lariat-driven circularization, intron pairing-driven circularization, or RNA-binding protein-driven circularization. circRNAs absorb microRNAs (miRNAs) to act as sponges for miRNAs and inhibit the transcription of the miRNA target gene [64]. miRNAs are short non-coding RNAs, which are not translated into proteins. miRNAs basically consist of approximately twenty bases and bind to mRNAs, leading to the degradation of mRNAs or the inhibition of translation from mRNA. Thus, miRNAs play a role in the intrinsic RNA silencing [65]. The 3′-untranslated region of PD-L1 contains the target sequence of miR-200 [66].

COUP-TF III, which is also known as nuclear receptor subfamily 2 group F member 6 (NR2F6) or v-erbA-related protein, is an orphan nuclear receptor [67,68]. Nr2f6 knockout mice exhibit a substantially higher expression of PD-1 and PD-L1. In addition, Nr2f6 knockout mice are resistant to the generation of sarcoma induced by methylcholanthrene and sarcoma transplantation [69]. Moreover, anti-PD-L1 antibody treatment reduces the tumor growth of Nr2f6 knockout mice with sarcoma transplantation [69,70]. In addition, the PD-L1 expression levels are increased in the CD4^+^ and CD8^+^ T cells from Nr2f6 knockout mice [69].

ER is also one of members of the steroid hormone nuclear receptor group [71]. Although ER is activated by ligands, such as estrogen, ER also functions in a ligand-independent manner [60,72,73]. The PD-L1 mRNA expression levels in ERα-positive breast cancer cells are lower compared to the PD-L1 mRNA expression levels in ERα-negative tumors from patients with breast cancer [74]. In addition, MCF7 cells, which are ER-positive but human epidermal growth factor receptor 2 (HER2)-negative cells, also express low levels of PD-L1 mRNA. Thus, ER negativity is proportional to high PD-L1 mRNA expression. Moreover, treatment with estradiol repressed the expression of PD-L1 mRNA [75]. In addition, the knockdown of ERα by small interfering RNA (siRNA) results in the upregulation of PD-L1 in MCF7 cells. However, knockdown of ERβ by siRNA did not upregulate the expression of PD-L1 in MCF7 cells [76]. The expression of ER is associated with the expression of IL-17E, which is also known as IL-25. IL-17E leads to a reduction in IL-17A, C, and F [77]. IL-17A enhances the expression of PD-L1 in triple-negative (ER-negative, PR-negative, and HER2-negative) breast cancer cells (MDA-MB-231 and SKBR-3 cells) via the phosphorylation of extracellular signal-regulated kinases [78]. Comparing the breast cancer cell lines, it is noted that the ER-positive breast cancer cell lines (MCF7 and T47D) do not express PD-L1, but the ER-negative breast cancer cell lines (BT-20, BT-549, HCC38, MDA-MB-231, and SKBR-3) do express PD-L1. However, AU565 and HCC1143, which are ER-negative breast cancer cell lines, do not express PD-L1 [75,78,79]. In addition, the treatment of MC38-colon-tumor-injected mice with estrogen and anti-PD-L1 antibody significantly reduced MC38 tumor growth compared to estrogen or anti-PD-L1 antibody alone [80].

Farnesoid X receptor (FXR) forms a heterodimer with RXR and the ligands for FXR are bile acids. FXR regulates various genes related to the metabolic, immune, and nervous systems [81]. The expression of FXR and PD-L1 in non-small cell lung cancer (NSCLC) and hepatocellular carcinoma cells is inversely correlated [82,83,84]. The knockdown of FXR by siRNA leads to the promotion of PD-L1 expression in NSCLC cells. In addition, FXR binds to the promoter region of the PD-L1 gene [83].

GR positively or negatively regulates the expression of a wide variety of genes [85,86]. The knockdown of GR by shRNA results in a reduction in PD-L1 in SU86.86 cells, which are pancreatic cancer cells. Moreover, the treatment of SU86.86 cells with dexamethasone induces the expression of PD-L1, and several glucocorticoid response elements have been found in the promoter region of PD-L1. In addition, the expression of GR is related to the expression of PD-L1 and a low survival rate in patients with pancreatic cancer [87]. However, treatment with dexamethasone leads to a decrease in the expression of PD-L1 in other cancer cell lines, including SGC-7901 (human gastric cancer cell line), MKN-45 (human gastric cancer cell line), SMMC-7721 (human hepatocarcinoma cell line), and BxPC3 (human pancreatic cancer cell line). Dexamethasone enhances the formation of GR and the STAT3 complex. The human PD-L1 gene is located at chr9:5450503-5470566. The GR/STAT3 complex binds to the binding sites of STAT3 in the promoter region of human PD-L1 (ch9:5449027-5449342), which is from −1.5 k to −1.2 kb in the promoter region of the human PD-L1 gene, and inhibits PD-L1 gene expression by STAT3 [88].

NUR77, which is also known as nuclear receptor subfamily 4 group A member 1 (NR4A1), is an orphan nuclear receptor. NUR77 plays an important role in the regulation of cell proliferation, apoptosis, and the metastasis of various tumors, including breast, colorectal, and liver cancers [89,90]. It has been reported that 1,1-Bis(3′-indolyl)-1-(p-hydroxyphenyl) methane (CDIM-8, which is also known as DIM-C-pPhOH) and 1,1-bis(3′-indolyl)-1-(3-chloro-4-hydroxy-5-methoxyphenyl)methane (Cl-OCH_3_) act as NR4A1 antagonists [91]. Treatment with CDIM-8 or Cl-OCH_3_ leads to a decrease in the expression of PD-L1 in breast cancer cell lines (Hs578T, SUM159PT, MDA-MB-231, and 4T1), a lung cancer cell line (A549), a colon cancer cell line (SW480), and a kidney cancer cell line (786-0). Moreover, the knockdown of NR4A1 by siRNA leads to the decreased expression of PD-L1 in MDA-MB-231 and 4T1 cells [92,93]. NR4A1 binds to the −0.2 kb promoter region of the human PD-L1 gene with SP1, and CDIM-8 and Cl-OCH_3_ inhibit the binding of NR4A1 to the promoter region of the human PD-L1 gene [92]. In addition, NR4A1 binds to the AP-1 binding site and prevents AP-1-induced gene expression by blocking transcription by AP-1 [94].

PPARγ forms a heterodimer with RXR and binds to the PPAR response elements (PPAREs). PPARγ regulates various genes associated with metabolism related to lipid, glucose, and cholesterol [95,96]. Treatment with PPARγ ligands, such as rosiglitazone and pioglitazone, induces the expression of PD-L1 in HT29 and HCT116 cells, which are gastrointestinal adenocarcinoma cell lines. Furthermore, PPARγ with the treatment of rosiglitazone binds to PPAREs in the proximal −2 kb promoter of the human PD-L1 gene [97]. Furthermore, PPARγ binds to PPREs in the PD-L1 promoter and treatment with rosiglitazone induces the expression of PD-L1 in tumor organoids from microsatellite-stable positive patients with colorectal cancer. Moreover, treatment with rosiglitazone and IFN-γ reduces the cell viabilities of tumor organoids in the presence of the wild-type anti-PD-L1 antibody. It is speculated that the wild-type anti-PD-L1 antibody blocks the PD-L1 induced by rosiglitazone and IFN-γ, but also bridges the Fc receptor on NK/T-like cells and PD-L1 on tumor organoids, promoting the attack of tumor organoids by NK/T-like cells [97]. In addition, treatment with GW9662, which is a PPARγ antagonist, suppresses the expression of PD-L1 in 3T3-L1-cell-induced adipogenesis [98].

RORγ, which is also known as retinoic-acid-related orphan receptor C (RORC), is an orphan nuclear receptor and plays an important role in the immune system [99,100,101]. The overexpression of RORγ in human 5637 and UC3 cells, which are bladder cancer cell lines, leads to a reduction in PD-L1 expression and the suppression of the PD-L1 promoter activity. RORγ binds to the ROR response element in the proximal −1.2 kb promoter of the human PD-L1 gene. In addition, the overexpression of RORγ represses the expression of the STAT family proteins, including STAT1 and STAT3. Moreover, the overexpression of RORγ leads to the phosphorylation of STAT3 and suppresses the binding activity of STAT3 [102].

Homologue of the drosophila tailless gene (TLX), which is also known as nuclear receptor subfamily 2 group E member 1 (NR2E1), is an orphan nuclear receptor [103]. However, a recent study demonstrated that oleic acid may be the ligand for TLX [104]. The expression of TLX is correlated with the expression of PD-L1 in glioma tissue in patients with glioma. In addition, the knockdown of TLX by shRNA leads to the repression of PD-L1 in the human glioblastoma cell line A1235. TLX binds to the proximal −122 to −90 promoter of the human PD-L1 gene [105].

VDR forms a heterodimer with RXR and binds to the VDR response elements (VDREs). VDR plays a crucial role primarily in the regulation of calcium homeostasis [106]. PD-L1 is upregulated by the 1,25-dihydroxyvitamin D treatment of human squamous cell carcinoma cells (SCC25 and SCC4) and human myeloid cells (THP-1 cells). Furthermore, VDR binds to the VDRE in the proximal −0.8 kb promoter region of the human PD-L1 gene in SCC25 cells and THP-1 cells that were differentiated into macrophages. In addition, the treatment of a co-culture of SCC25 cells and T cells from a healthy donor with 1,25-dihydroxyvitamin D reduces T cells from the healthy donor, and anti-PD-L1 antibody partially or completely prevents the reduction in T cells [107].

## 5. Concluding Remarks

The function of PD-L1 as a ligand to PD-1 has been extensively studied. PD-L1 expression in cancer cells enables them to escape the immune system. Thus, preventing cancer cells from escaping the immune system requires the inhibition of PD-1 and PD-L1 interactions, the inhibition of the PD-1 signaling cascade, and the inhibition of PD-1 or PD-L1 expression. Antibody drugs such as anti-PD-1 and PD-L1 antibodies have been developed to inhibit PD-1 and PD-L1 interactions. In addition, combination therapy using anti-PD-1 and PD-L1 antibodies with other drugs is necessary to improve the response rate of cancer treatment, and substances that inhibit PD-1 signaling or inhibit PD-1 or PD-L1 expression may be potential candidates. Several transcription factors involved in the regulation of PD-L1 expression have been identified. However, nuclear receptors can also positively or negatively regulate PD-L1 expression. Further study on these nuclear receptors and the elucidation of PD-L1 expression mechanisms by these nuclear receptors will lead to the development of new cancer therapies.
